# Frequent low dose alcohol intake increases gastric cancer risk: the Health Examinees-Gem (HEXA-G) study

**DOI:** 10.20892/j.issn.2095-3941.2021.0642

**Published:** 2022-04-28

**Authors:** Hwi-Won Lee, Dan Huang, Woo-Kyoung Shin, Katherine de la Torre, Minkyo Song, Aesun Shin, Jong-Koo Lee, Daehee Kang

**Affiliations:** 1 Department of Biomedical Sciences, Seoul National University Graduate School, Seoul 03080, Korea; 2 Department of Preventive Medicine, Seoul National University College of Medicine, Seoul 03080, Korea; 3 Integrated Major in Innovative Medical Science, Seoul National University Graduate School, Seoul 03080, Korea; 4 Department of Family Medicine, Seoul National University Hospital, Seoul 03080, Korea

**Keywords:** Gastric cancer, alcohol consumption, drinking behavior, prospective cohort, Health Examinees-Gem (HEXA-G) study

## Abstract

**Objective:**

Epidemiological studies indicate that alcohol increases gastric cancer (GC) risk, yet most studies have focused on heavy alcohol intake, leaving other factors understudied. A comprehensive investigation of the effects of the frequency and amount of alcohol intake may help elucidate the GC risk associated with drinking behavior.

**Methods:**

The Health Examinees-Gem (HEXA-G) study, a community-based large-scale prospective cohort study, enrolled Korean adults 40–69 years of age between the years 2004 and 2013. Incident GC cases were identified through linkage to Korea Central Cancer Registry data until December 31, 2017. Self-reported questionnaires were used to survey alcohol consumption-related factors (duration, frequency, amount, and type of alcoholic beverages). The frequency and amount of alcohol consumption were combined to explore GC risk according to 4 drinking patterns: “infrequent-light”, “frequent-light”, “infrequent-heavy”, and “frequent-heavy”. We used Cox proportional hazard models to estimate the adjusted hazard ratios (HRs) and 95% confidence intervals (CIs), and investigate the relationship between alcohol intake and GC incidence.

**Results:**

A total of 128,218 participants were included in the analysis. During an average follow-up period of 8.6 years, 462 men and 385 women were diagnosed with GC. In men, current drinkers showed a 31% greater risk of GC than non-drinkers (HR 1.31, 95% CI 1.03–1.66), whereas no significant association was observed in women. In men, GC risk was associated with a higher frequency (*P* trend 0.02) and dose of ethanol intake in grams (*P* trend 0.03). In men, the “frequent-light” (≥5 times/week and <40 g ethanol/day) drinking pattern was associated with a 46% greater risk of GC (HR 1.46, 95% CI 1.02–2.07) than the “infrequent-light” pattern (<5 times/week and <40 g ethanol/day).

**Conclusions:**

This study suggests that frequent intake of alcohol, even in low quantities per session, increases GC risk. Further research is warranted to evaluate the relationship between alcohol and GC in detail.

## Introduction

In most populations, the incidence and mortality rates for gastric cancer (GC) have been steadily declining, but the incidence rates remain high in East Asian countries^1^. The considerable geographic variations in incidence and mortality rates indicate that environmental and lifestyle factors play important roles in the etiology of GC^2^.

According to the World Cancer Report, the epidemiological evidence of an association between alcohol consumption and stomach is conflicting^3^. Alcohol has been considered a group 1 carcinogen by the International Agency for Research on Cancer Monographs Programme since 1988, and alcohol consumption has been causally associated with cancers of the oral cavity, esophagus, liver, colorectum, and breast in women^4–8^. However, for GC, the evidence of a link to alcohol consumption remains limited, and a weaker dose-risk relationship has been observed^4,9^.

The 2018 Continuous Update Project by the World Cancer Research Fund has reported that an intake of approximately 45 grams or more of pure alcohol per day, equivalent to 3 drinks, is a probable cause of GC^10^. However, no conclusions were made for intakes below 45 grams of ethanol in the meta-analysis. Likewise, numerous epidemiological studies have supported the harmful effects of heavy episodic drinking or binge drinking^11^, yet the effects of light to moderate levels of alcohol drinking remain to be clarified, owing to inconsistent results^12–15^. Additionally, Deng et al.^16^, in a recent meta-analysis exploring the association between alcohol consumption and increased gastric cancer risk, have reported a significant association only in case-control studies but not cohort studies. Assessment of drinking patterns, such as a range of intake levels, should be explored with respect to drinking frequency, to better elucidate the relationship between alcohol intake and GC development. These findings could aid in identifying preventable real-world measures associated with drinking behavior.

Using a large-scale, prospective cohort, this study aimed to investigate the sex-specific association of alcohol drinking patterns, specifically frequency and quantity, with the risk of GC in a population with a high incidence rate of GC.

## Materials and methods

### Study population

The Health Examinees-Gem (HEXA-G) study is a subset of the larger HEXA study, one of the 6 prospective cohorts in the Korean Genome and Epidemiology Study (KoGES)^17^. The HEXA study is a large-scale, population-based prospective cohort of Korean adults 40–69 years of age who visited health examination centers in 38 regions from 2004 to 2013^18^. The HEXA-G data used in this study followed additional eligibility criteria, excluding the following: 1) sites that participated in the pilot study only from 2004 to 2006; 2) sites that did not meet the HEXA standards for biospecimen quality control; 3) sites that participated in the study for <2 years^19,20^. To date, 139,273 participants in the HEXA-G study completed the initial follow-up survey until 2017.

**Figure 1:**
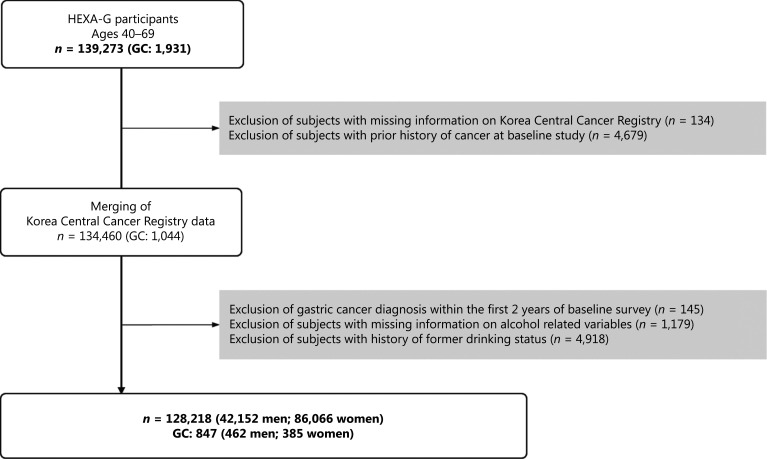
Flow chart of the study population. HEXA, Health Examinees Study; GC, gastric cancer.

In this study, we excluded HEXA-G participants with missing information in the Korea Central Cancer Registry (KCCR; *n* = 134) and those with a history of cancer in the baseline study (*n* = 4,679). Participants with missing information for alcohol-related variables (*n* = 1,179) and former drinkers (*n* = 4,918) were further excluded (**Figure 1**). We also excluded participants who were diagnosed with GC within the first 2 years of the baseline survey (*n* = 145) to minimize reverse causality effects. We ultimately included 128,218 participants (42,152 men and 86,066 women) in the final analysis.

The HEXA-G study protocol was approved by the Institutional Review Board (IRB) of Seoul National University Hospital (IRB number E-2009-117-1159) and the Ethics Committee of KoGES of the Korea National Institute of Health (IRB number 2014-08-02-3C-A).

### Gastric cancer case ascertainment

Incident GC cases were identified through linkage to the data provided by the KCCR, which is part of the National Cancer Center of Korea. Currently, more than 190 hospitals participate in the KCCR, which includes more than 90% of the new cancer cases in the Republic of Korea, through a collection of nationwide data including regional cancer registries^21,22^. Through collaboration with the Korea Disease Control and Prevention Agency, we obtained data on cancer incidence until December 31, 2017 *via* resident registration numbers uniquely assigned to each resident in the Republic of Korea. The primary outcome was defined as the first occurrence of an International Classification of Diseases, 10th Revision, code for GC (C16.0–16.9) after the baseline study. In a secondary analysis, we further classified GC into anatomical subsites: cardia (C16.0) and non-cardia (C16.1–16.9).

### Alcohol consumption

Alcohol consumption was assessed with a self-administered questionnaire, which was collected during the baseline survey. Participants were surveyed regarding their alcohol consumption status during the past year, and whether they were non-drinkers, or former or current drinkers. Because we could not identify people who might have ceased drinking because of health problems, only the results for current drinkers were included in the analysis. Participants were also queried regarding the duration (years) and frequency (per week or month) of drinking; type of alcoholic beverages [e.g., *soju* (Korean liquor distilled or diluted from rice, wheat, or sweet potato), beer, *makgeolli* (Korean alcoholic beverage fermented from rice and other cereals), strong spirits, wine, and *cheongju* (Korean clear, refined rice wine)]; and average amount of alcohol intake per drinking session. We used the equation provided by the Ministry of Food and Drug Safety and the Korea Health Promotion Institute to derive the amount of pure alcohol or ethanol in different types of alcoholic beverages^23–25^:

$$\text{ethanol}\;\text{(g) = }\frac{\text{intake quantity (mL)}\times \text{alcohol by volume (\%)}\times \text{alcohol density at room temperature }(0.79)}{100}$$

For information on the percentage of alcohol by volume, we referred to basic consumer information on the labels of different types of beverages: *soju*, 19%; beer, 5%; *makgeolli*, 6%; strong spirits, 43%; wine, 13%; and *cheongju*, 13%. To determine the average daily intake of ethanol, we multiplied the average drinking frequency per day by the amount of ethanol intake per session.

We classified the duration of alcohol consumption into quintiles for men (non-drinkers, ≤20, 21–30, 31–40, and ≥41 years) and quartiles for women (non-drinkers, ≤10, 11–20, and ≥21 years). The frequency of alcohol consumption was categorized into quintiles for men (non-drinkers, ≤1, 2–3, 4, and ≥5 times per week) and quartiles for women (non-drinkers, <1, 1, and ≥2 times per week). The amount of alcohol consumed was divided into quartiles for both men (non-drinkers, <10, 10–30, and >30 g ethanol per week) and women (non-drinkers, <10, 10–20, and >20 g ethanol per week). Additionally, we combined the alcohol frequency and amount to examine the alcohol consumption patterns associated with GC risk in men. In the combined analysis, the amount of pure alcohol intake in grams was assessed according to the World Health Organization recommendation for low-risk drinking in men (40 g/day)^26^. For frequency, we used the median weekly alcohol intake (5 times/week) among people who had ever consumed alcohol in our study. Consumption patterns were therefore classified into the following 5 groups: non-drinkers, and “infrequent-light” (<5 times/week and <40 g ethanol/day), “infrequent-heavy” (<5 times/week and ≥40 g ethanol/day), “frequent-light” (≥5 times/week and <40 g ethanol/day), and “frequent-heavy” (≥5 times/week and ≥40 g ethanol/day) drinkers.

### Covariates

We considered factors such as age (continuous), sex, family history of GC, educational level (lower than or including middle school/high school diploma/higher than a college degree), marital status (married/cohabitation), smoking status (never/ever smoked), physical activity (regular/irregular), body mass index (BMI, continuous in kg/m^2^), and total energy intake (continuous in kcal/day) as covariates. This information was collected by trained interviewers through anthropometric measurements and structured questionnaires including socio-demographics, medical history, family history, and lifestyle factors, and dietary intake in the baseline survey.

### Statistical analysis

We used Student’s t-test and chi-square test for continuous variables and categorical variables, respectively, to test the differences in the distribution of epidemiologic factors between alcohol drinkers and non-drinkers. To explore sex-associated differences in epidemiologic risk factors, we performed separate analyses for men and women. We used Cox proportional hazard models to estimate the adjusted hazard ratios (HRs) and 95% confidence intervals (CIs), and to explore the associations between GC incidence and various alcohol-related factors; the duration, frequency, and quantity of drinking; and combined drinking patterns. For multivariable HRs, we adjusted for family history of GC, educational status, marital status, smoking status (men only), regular physical activity, BMI, and total energy intake. Smoking status was adjusted in only men, owing to the low prevalence of having ever smoked among women. Alcohol intake in increments of 10 g was also used for risk estimation.

The follow-up period for the study participants was defined as the period between the date at which the baseline study was completed and the dates of GC diagnosis, loss to follow-up, or the final follow-up date (December 31, 2017), whichever came first. We further performed analyses of alcohol consumption according to GC anatomical subsites. A sensitivity analysis was conducted by using baseline incident cases of GC. All statistical analyses were performed in SAS software version 9.4 (SAS Institute, Cary, NC, USA), and *P* values less than 0.05 were considered statistically significant.

## Results

During an average follow-up period of 8.6 (±2.1) years for all participants, 462 (1.1%) men and 385 (0.5%) women were identified as having GC.

**Table 1** shows the baseline characteristics of the HEXA-G study population included in the analysis. The proportion of current drinkers was 78.7% for men and 31.7% for women, respectively. Among men, the current drinkers were younger, had attained higher educational status, were more likely to have ever smoked, were physically inactive, and had higher BMI and total energy intake than non-drinkers. Women who were current drinkers were younger, had attained higher educational status, were single, were physically inactive, and had higher BMI and total energy intake than non-drinkers.

**Table 1:** Baseline characteristics of the HEXA-G study population included in the alcohol consumption analysis (*n* = 128,218)^†^

Characteristics	Men (*n* = 42,152)				*P* value*	Women (*n* = 86,066)				*P* value*
	Non-drinker (*n* = 8,968)		Drinker (*n* = 33,184)			Non-drinker (*n* = 58,825)		Drinker (*n* = 27,241)		
	*n*	(%)	*n*	(%)		*n*	(%)	*n*	(%)	
Follow-up year (mean ± SD)	8.7	(2.0)	8.7	(2.0)	0.640	8.8	(2.1)	8.7	(2.1)	<0.001
Age (mean ± SD)	55.0	(8.4)	52.7	(8.3)	<0.001	53.4	(7.8)	49.6	(7.0)	<0.001
Family history of GC	786	(8.8)	2,670	(8.1)	0.068	5,053	(8.6)	2,239	(8.2)	0.192
Education					<0.001					<0.001
≤Middle school	2,044	(22.8)	6,681	(20.1)		22,566	(38.4)	8,681	(31.9)	
High school diploma	3,525	(39.3)	13,728	(41.4)		24,264	(41.3)	12,764	(46.9)	
≥College degree	3,251	(36.3)	12,369	(37.3)		11,148	(19.0)	5,490	(20.2)	
Married/cohabitation	8,401	(93.7)	31,147	(93.9)	0.005	51,203	(87.0)	23,337	(85.7)	<0.001
Ever smokers^‡^	4,887	(54.5)	25,566	(77.0)	<0.001	1,146	(2.0)	1,882	(6.9)	<0.001
Regular exercisers	4,165	(46.4)	13,936	(42.0)	<0.001	29,319	(49.8)	12,777	(46.9)	<0.001
BMI (kg/m^2^, mean ± SD)	23.7	(4.5)	23.7	(1.1)	<0.001	23.7	(2.5)	23.7	(4.1)	<0.001
Total energy intake (kcal, mean ± SD)	563.7	(115.3)	1,871.0	(559.7)	<0.001	1,700.36	(568.0)	1,736.64	(605.2)	<0.001

HEXA-G, Health Examinees-Gem study; GC, gastric cancer; BMI, body mass index.

*Student’s t-test for continuous variables; chi-square test for categorical variables.

^†^Data for continuous variables are presented as mean ± standard deviation (SD); all other variables are presented as *n* (%).

^‡^Ever smokers is defined as lifetime smokers with ≥20 pack-years.

**Table 2** shows the sex-specific hazard ratios and 95% CIs for GC according to drinking status, and the duration, frequency, and amount of weekly intake of ethanol. Among men, current drinkers had a 31% higher risk of GC than non-drinkers (HR 1.31, 95% CI 1.03–1.66), but no association was observed in women (HR 1.00, 95% CI 0.79–1.26). No significant association between the duration of alcohol drinking and GC risk was observed in either men or women. Compared with not drinking, a higher frequency of alcohol drinking was significantly associated with GC risk (*P* trend 0.02). In fact, men in the highest drinking frequency category (≥5 times/week) had a 62% greater risk of GC than non-drinkers (HR 1.62, 95% CI 1.17–2.25). In terms of intake of weekly ethanol in grams, GC risk showed a significant dose-response relationship among men, in drinkers compared with non-drinkers (<10 g/week: HR 1.25, 95% CI 0.96–1.63; 10–30 g/week: HR 1.37, 95% CI 1.03–1.83; >30 g/week: HR 1.47, 95% CI 1.08–1.99; *P* trend 0.03). No factors relating to alcohol intake, or the frequency and amount of ethanol consumed, were significantly associated with GC risk in women.

**Table 2:** Associations between gastric cancer and alcohol consumption status, and the duration, frequency, and amount of ethanol consumed

Variables	Men (*n* = 42,152)							Variables	Women (*n* = 86,066)						
	*n*	Person-years	GC	(%)	HR^†^	95% CI			*n*	Person-years	GC	(%)	HR^†^	95% CI	
**Status**								**Status**							
Non-drinker	8,968	77,924	87	(1.0)	1.00	Ref		Non-drinker	58,825	515,102	283	(0.5)	1.00	Ref	
Drinker	33,184	287,973	375	(1.1)	1.31	1.03	1.66	Drinker	27,241	235,840	102	(0.4)	1.00	0.79	1.26
**Duration**								**Duration**							
Non-drinker	8,968	77,924	87	1.0	1.00	Ref		Non-drinker	58,825	515,102	283	0.5	1.00	Ref	
≤20 years	7,718	67,077	59	0.8	1.39	0.99	1.97	≤10 years	3,448	31,250	15	0.4	1.12	0.66	1.89
>20 years	24,782	214,205	311	1.3	1.32	1.03	1.68	>10 years	22,331	189,090	82	0.4	1.01	0.78	1.30
*P* trend					0.03			*P* trend					0.93		
**Frequency**								**Frequency**							
Non-drinker	8,968	77,924	87	(1.0)	1.00	Ref		Non-drinker	58,825	515,102	283	(0.5)	1.00	Ref	
≤1 time/week	9,856	85,099	113	(1.2)	1.32	1.00	1.75	<1 time/week	7,467	63,972	27	(0.4)	0.94	0.63	1.39
2–3 times/week	11,148	96,494	111	(1.0)	1.19	0.89	1.58	1 time/week	11,746	101,817	47	(0.4)	1.09	0.80	1.50
4 times/week	7,056	60,619	78	(1.1)	1.35	0.99	1.85	≥2 times/week	6,817	57,811	22	(0.3)	0.94	0.60	1.45
≥5 times/week	4,194	36,152	64	(1.5)	1.62	1.17	2.25	*P* trend					0.41		
*P* trend					0.02										
**Amount of ethanol** ^‡^								**Amount of ethanol** ^‡^							
Non-drinker	8,968	77,924	87	(1.0)	1.00	Ref		Non-drinker	58,825	515,102	283	(0.5)	1.00	Ref	
<10 g/week	14,373	123,873	159	(1.1)	1.25	0.96	1.63	<10 g/week	7,990	68,661	32	(0.4)	1.00	0.69	1.44
10–30 g/week	10,315	89,243	118	(1.1)	1.37	1.03	1.83	10–20 g/week	8,972	77,883	36	(0.4)	1.09	0.77	1.56
>30 g/week	7,602	65,621	91	(1.2)	1.47	1.08	1.99	>20 g/week	9,086	77,215	28	(0.3)	0.92	0.62	1.37
*P* trend					0.03			*P* trend					0.49		
Continuous, per 1 SD					1.00	1.00	1.00	Continuous, per 1 SD					0.87	0.65	1.17

GC, gastric cancer; HR, hazard ratio; CI, confidence interval; SD, standard drink.

^†^Adjusted for education, smoking status, marital status, family history of gastric cancer, exercise, BMI, and total energy intake.

^‡^Amount of ethanol refers to the amount of pure alcohol found in all alcohol beverages consumed by study participants, converted to grams per week.

**Table 3** presents results of the association of both the weekly frequency and daily amount of pure alcohol consumed with the development of GC in men. We combined alcohol frequency and amount to comprehensively assess weekly alcohol drinking patterns among men and obtain greater insight. Compared with the “infrequent-light” group [those who drank less frequently (<5 times/week) and consumed less than 40 g of ethanol per day], the “infrequent-heavy” group [those who drank equally less frequently but had higher daily ethanol intake (>40 g)] and the “frequent-heavy” group [those who drank more often (≥5 times/week) with higher daily ethanol intake (40 g)] did not show significantly higher risk of GC. However, for the “frequent-light” group [those who consumed less than 40 g of ethanol a day but drank more often (≥5 times/week)], the risk of GC incidence exceeded 45% (HR 1.45, 95% CI 1.02–2.07). Among non-drinkers, the risk of GC was statistically significantly lower than that of the reference group (HR 0.78, 95% CI 0.61–0.99).

**Table 3:** Association between alcohol consumption patterns and gastric cancer in men

Consumption pattern	Frequency	Amount of ethanol	*n*	Person-years	GC	(%)	HR^†^	95% CI	
Non-drinker			8,968	77,924	87	(1.0)	0.78	0.61	0.99
Infrequent-light	<5 times/week	<40 g ethanol/day	25,884	223,795	282	(1.1)	1.00	Ref	
Infrequent-heavy	<5 times/week	≥40 g ethanol/day	1,085	9,293	12	(1.1)	1.14	0.64	2.03
Frequent-light	≥5 times/week	<40 g ethanol/day	1,959	16,689	35	(1.8)	1.46	1.02	2.07
Frequent-heavy	≥5 times/week	≥40 g ethanol/day	2,173	18,964	27	(1.2)	1.04	0.70	1.54

GC, gastric cancer; HR, hazard ratio; CI, confidence interval.

^†^Adjusted for education, smoking status, marital status, family history of gastric cancer, exercise, BMI, and total energy intake.

In a supplementary analysis on men, we investigated the association between GC risk and different alcoholic beverages, beer and soju, and found that soju consumption was significantly correlated with a higher weekly frequency and daily amount of ethanol intake (*P* trend 0.03 for weekly frequency, *P* trend 0.04 for daily ethanol intake in grams) (**Supplementary Table S1**). Other beverage types were consumed in amounts too small to be analyzed as separate drink types and therefore were excluded. In analysis of anatomical subsites, no significant association was observed between gastric cardia cancer and alcohol intake in men; in women, the numbers were too small to be analyzed (**Supplementary Table S2**). For non-cardia gastric cancer, a significant direct relationship was observed between GC risk and the weekly frequency and weekly amount of ethanol in men (*P* trend 0.003 for weekly frequency, *P* trend 0.006 for weekly ethanol intake in grams) (**Supplementary Table S3**).

## Discussion

In this prospective study of Korean men and women, alcohol consumption factors were significantly associated with the risk of GC in men, which showed a linear increase with the weekly frequency and weekly amount of ethanol. However, among women, none of the alcohol-related factors showed significant relationships with GC incidence. In an analysis including former drinkers, we observed similar findings, in which alcohol-related factors were significantly associated with GC only in men (**Supplementary Table S4**). Moreover, the significant association was observed for the total population, when both sexes were combined (**Supplementary Table S5**). The results in the total population might have been due to the high number of GC cases in men. When the 2 factors were combined, a “frequent-light” drinking pattern, defined as frequent intake of low dose of daily alcohol, was prominently associated with GC risk.

We observed that a combined drinking pattern comprising consumption frequency and amount was an important indicator of GC risk. Additional analysis considered the effect of drink duration with combined drink patterns in men by stratification of alcohol intake duration (<21 years and ≥21 years). In contrast to the frequency and amount, the duration of alcohol consumption was not observed to be a significant factor of GC risk in men (**Supplementary Table S6**). These findings further supported our results, highlighting the importance of understanding GC risk factors through a descriptive approach considering behavioral dimensions of alcohol use. Recent studies have provided evidence that light consumption at ≤1 drink a day (12.5 g ethanol/day) is associated with adverse effects^27,28^, and other studies have report that the risk of cancer due to high drinking frequency is similar to that of heavy drinking^12^. Although these findings are similar to our results, they provide evidence for cancers other than GC. In terms of drinking patterns, only one study’s results are concordant with our findings^29^. This latest Korean research further indicates that the frequency of alcohol intake, rather than quantity, is a more important risk factor for gastrointestinal (GI) cancers including GC^29^. Nonetheless, further research is warranted to thoroughly explore and evaluate alcohol drinking patterns and GC risk.

Our results regarding frequency and quantity as independent factors are consistent with previous findings^30–35^. A cohort study of Korean men has reported a 20% increased risk of GC associated with alcohol consumption of ≥25 g per day (aRR 1.20, 95% CI 1.10–1.40)^31^. In accordance with our study results, the association was significant and dose dependent (*P* trend 0.0001). A Lithuanian study has reported that after 30 years of follow-up, both frequency and quantity of alcohol intake were associated with GC risk in men. Moreover, this prospective study found a 100% increase in GC risk with the highest drinking frequency of 2–7 times per week (HR 2.00, 95% CI 1.04–3.82) and a 90% greater risk of GC in association with a weekly intake amount of ≥100 g ethanol (HR 1.90, 95% CI 1.13–3.18), as compared with occasional drinking of 0.1–9.9 g ethanol per week^32^. In the recently updated World Cancer Research Fund report, when stratified by sex, outcome, and geographical region, the meta-analysis showed a significantly elevated risk of GC in men and in Asian cohorts^10^. The lack of association in women might have been due to the low number of current drinkers. Considering the nature of the survey method used in the baseline study, misclassification of drinking status among women might have been a possibility, but the prevalence of alcohol consumption among women in our study (31.7%) was concordant with those in the KNHANES, which ranged from 28.4% to 34.2% between 2005 and 2013^19,26,36^. Moreover, several previous studies have shown that alcohol consumption increases the serum levels of female sex hormones, which may protect against GC^37,38^.

Regarding the null association observed among “frequent-heavy” drinkers, we reflect on some of the limitations offered in alcohol-related studies. According to some epidemiologists’ studies on drinking, types of selection bias that can occur in alcohol-related research include sick quitter bias, healthy survivor bias, cohort age, and poor baseline health status^39^. Alcohol-related risk is relatively higher among people in the younger age range of 20–49 years^40^; thus, the risk relationship is minimized if studies include older participants rather than drinkers of all ages^41^. In fact, our study participants’ ages ranged from 40 to 69 years at enrollment; according to prior studies on alcohol, this characteristic increases the likelihood of drinkers becoming former drinkers during follow up^42^. Moreover, Naimi et al.^40^ have argued that the age of the cohort might influence the number of events, such as deaths attributable to alcohol intake. Over-representation of older age groups among cohort studies has been reported to lead to underestimation of drinkers relative to non-drinkers^39^. In many developed countries, including the United States, approximately 90% of drinkers have already begun drinking at the age of 21^43^. In Korea, the average initiation age of drinking is 21.3 years, an age much younger than the common age of cohort enrollment; moreover, the age group of 30–49 years has been observed to consume the largest amount of alcohol^44^. Therefore, the average age of many cohort participants might not be representative of that of drinkers. Despite these potential biases, we conducted an analysis that combined the frequency and amount of alcohol consumption to better elucidate which alcohol-related factors are closely correlated with GC risk. Considering the potential biases that may arise from alcohol-related studies, caution is necessary in interpreting our results.

Several mechanisms may provide understanding of the association between alcohol and GC. A potential mechanism of increased GC risk associated with recurrent or chronic exposure to alcohol by frequent drinking may involve the combined actions of ethanol, acetaldehyde, and nitrosamines in the gastric mucosa. Notably, alcohol has been shown to permanently damage DNA strands in cells and to inhibit DNA repair processes, particularly through acetaldehyde, the immediate product of alcohol metabolism^45^. Acetaldehyde, a carcinogenic metabolite of alcohol, is associated with upper GI tract cancer *via* direct mucosal damage^9^. The constant exposure to acetaldehyde in saliva and gastric juice may cumulatively impair the GI tract, thus depriving the body of the time to heal^9^.

Alcohol consumption can also decrease folate levels, inhibit key enzymes in one-carbon metabolism, and hinder the activity and expression of DNA methyltransferases, all of which lead to aberrant patterns of DNA methylation^46^. Both chronic and heavy use of alcohol induces the expression of cytochrome P450 2E1 (CYP2E1) in the human liver and rat gastrointestinal mucosa^47,48^. Thus, alcohol-induced CYP2E1 could contribute to the formation of reactive oxygen species in the gastrointestinal tract and to the activation of procarcinogens, such as nitrosamines, that may be present in alcoholic beverages^47^. The volatile N-nitroso compound N-nitrosodimethylamine, found in beer, whiskey, and other hard liquors, has been suggested to be a potent carcinogen in animals^49^.

In a less direct manner, alcohol also acts as a solvent enhancing the penetration of carcinogens into cells^50^. Furthermore, alcohol may interfere with retinoid metabolism, thus adversely affecting cellular growth, cellular differentiation, and apoptosis^49^. Regular use of alcohol may also lead to deficiencies in essential nutrients that affect DNA processing pathways and susceptibility to carcinogenesis^45,51^.

In addition, some genetic variations are associated with an increased risk of cancer development due to alcohol consumption^8,27^. ALDH2*2, a variant associated with acetaldehyde oxidizing enzymes, which is prevalent among Asians, promotes acetaldehyde accumulation in peripheral blood after the intake of even small amounts of alcohol, thereby causing mutagenic effects^27^. Studies have reported that the overall risk of upper GI tract cancer is elevated among East Asians, among whom aldehyde dehydrogenase deficiency is common^52,53^. Meta-analyses of alcohol-related cancers have reported higher relative risk (RR) in Asian than Western populations for similar doses of alcohol intake^10,54^. With differences in gene status and the incidence of GC, caution is necessary in assessing the generalizability of our findings to a non-Korean population.

Some inevitable limitations in our study should be noted. First, alcohol intake information was based on self-reporting during the baseline survey, thus potentially allowing for misclassification, underreporting, and omission of information regarding changes regarding alcohol consumption habits during follow-up^22^. However, the nature of our prospective cohort study minimized the possibility of recall bias. Hence, some measurement errors that persisted might have been nondifferential. Moreover, the questionnaires used in the HEXA survey, including that for alcohol consumption, are sufficiently detailed to provide information from each participant by exploring various aspects such as the frequency, amount, duration, and type of alcoholic beverages. By including the differing alcohol content of various alcoholic beverages in our calculations, we were able to analyze more comprehensive data regarding the quantity of alcohol intake. These questions were derived from validated surveys and standards used in national and organizational sectors, such as the KNHANES as well as the World Health Organization^17,18^. Second, our data lack information on *H. pylori* infection, a strong risk factor for GC^23–24^. Approximately one-third of adults are infected with *H. pylori* worldwide^55^; in Korea, the infection prevalence has been estimated to be approximately 44% in the adult population^56^. Despite this high prevalence, the relationship between *H. pylori* infection and GC in Korea lacks support from epidemiological data. A nested case-control study in Korea has found no direct association between *H. pylori* infection GC^57^. Moreover, a Chinese study has reported that the adjustment of *H. pylori* does not substantially change the association between alcohol consumption and GC risk^35^. In fact, several studies have reported that alcohol intake is independently associated with GC^58–61^. Results regarding the prevention of GC after eradication of the infection remain ambiguous^62^. Third, although we attempted to control for important confounders, we cannot rule out the potential effects of residual confounders, including dietary factors such as salty food, tea, and garlic consumption, which could not be accounted for. However, we attempted to control for important confounders by taking potential confounding factors into account in the main analyses. Moreover, we investigated tea drinkers in a supplementary analysis. Because consumption of only green tea was available in our data, we conducted a subgroup analysis in men and women who consumed green tea and found that the results were in line with those of our main analysis (**Supplementary Table S7**). Regarding salty food intake, a recent systematic review and meta-analysis of prospective cohort studies has indicated that the risk of GC is increased by 12% per 5 g/day increase in dietary salt intake or 5% per 10 g/day increase in alcohol consumption, and the 2 factors are independent of each other^63^. Moreover, a Korean prospective study has suggested that dietary salt intake has an independent effect on the occurrence of GC^64^. Finally, because only 40 cases of gastric cardia cancer were identified in our study, our results could not provide sufficient evidence to understand the relationships specific to anatomical subsites.

Despite these limitations, the strength of our study lies in that it is one of the largest community-based prospective cohorts in Korea, which explored crucial factors involved in alcohol drinking among the middle-aged and older population. In addition, this study reflects sex differences in epidemiologic risk factors, because we performed separate analyses for men and women. We also excluded GC cases that occurred within the first 2 years after the baseline survey, to prevent reverse causation, thus resulting in a more conservative association between alcohol consumption and GC. The inclusion of various factors potentially associated with alcohol-related behaviors in this large-scale cohort analysis enabled us to identify behavioral patterns whose associations with gastric cancer requires require further exploration. Our findings may help provide a context for public health recommendations and programs aimed at not only high-risk drinkers but also at the entire drinking population, to effectively decrease the health burden associated with alcohol drinking in non-Western regions such as Korea.

In conclusion, higher alcohol consumption was significantly associated with GC among Korean men. In terms of drinking patterns, “frequent-light” drinkers were at risk of GC: frequent intake of alcohol, even in low quantities per drinking session, was a significant risk factor for GC. Further research on gene and environmental interactions among different ethnicities is warranted to provide a more detailed evaluation of alcohol drinking and GC risk.
