# Supplementary material for: Frequent low dose alcohol intake increases gastric cancer risk: the Health Examinees-Gem (HEXA-G) study
Source: Cancer Biol Med. 2022 Apr 28;19(8):1224–34. doi: 10.20892/j.issn.2095-3941.2021.0642 (PMC9425184; doi:10.20892/j.issn.2095-3941.2021.0642)
Supplement: Supplementary file 1 [file cbm-19-1224-s001.pdf]

Supplemetary materials

Table S1 Hazard ratios and 95% confidence intervals of gastric cancer according to drink type

Variables	Men 42,152 (GC = 462)						
	Number of participants	Person-years	GC	(%)	HR†	95% CI	
**Beer**							
**Drink frequency (times/week)**							
Non-drinker	8,968	77,924	87	(1.0)	1.00	Ref	
<1	9,792	84,031	94	(1.0)	1.25	0.92	1.70
1 or 2	3,395	29,192	37	(1.1)	1.47	0.99	2.19
≥3	906	7,739	9	(1.0)	1.18	0.59	2.36
P trend					0.514		
**Ethanol intake (g ethanol/day)**							
Non-drinker	8,968	77,924	87	(1.0)	1.00	Ref	
≤10	10,654	91,009	102	(1.0)	1.01	0.81	1.27
>10	3,222	27,523	38	(1.2)	1.31	0.93	1.84
P trend					0.126		
Continuous, per 1 SD					1.01	0.92	1.11
**Soju**							
**Drink frequency (times/week)**							
Non-drinker	8,968	77,924	87	0.97	1.00	Ref	
<1	14,949	129,331	161	1.08	1.26	0.99	1.60
1 or 2	10,586	91,884	117	1.11	1.29	1.00	1.68
≥3	4,351	37,648	64	1.47	1.47	1.08	2.01
P trend					0.028		
**Ethanol intake (g ethanol/day)**							
Non-drinker	8,968	77,924	87	(1.0)	1.00	Ref	
<10	16,243	140,653	182	(1.1)	1.26	0.99	1.59
>10	13,643	118,210	160	(1.2)	1.37	1.07	1.75
P trend					0.037		
Continuous, per 1 SD					1.01	0.97	1.05

GC, gastric cancer; HR, hazard ratio; CI, confidence interval; SD, standard drink.
†Adjusted for education, smoking status, marital status, family history of gastric cancer, exercise, BMI, and total energy intake.

Table S2 Hazard ratios and 95% confidence intervals of gastric cardia cancer (n = 41,406) according to alcohol consumption in men

Variables	Number of participants	Person-years	GC	(%)	HR[†]	95% CI	
**Drink status**							
Never	8,779	71,236	‡	(17.4)	1.00	Ref	
Ever	32,627	267,598	19	(82.6)	1.34	0.44	4.02
**Drink duration (years)**							
Non-drinker	8,779	71,236	‡	(17.4)	1.00	Ref	
≤20 years	7,626	64,052	‡	(17.4)	1.62	0.37	7.15
21–30 years	13,088	109,816	5	(21.7)	1.00	0.25	4.01
31–40 years	8,508	66,900	6	(26.1)	1.3	0.36	4.74
≥41 years	2,727	20,583	‡	(17.4)	1.94	0.45	8.40
P trend					0.54		
**Drink frequency (times/week)**							
Non-drinker	8,779	71,236	‡	(17.4)	1.00	Ref	
≤1 time/week	9,694	78,897	10	(43.5)	2.71	0.84	8.73
2–3 times/week	10,975	90,377	6	(26.1)	1.43	0.40	5.17
4 times/week	6,937	56,339	‡	(8.7)	0.75	0.13	4.17
≥5 times/week	4,105	33,151	‡	(4.4)	0.54	0.06	4.90
P trend					0.15		
**Ethanol intake (g ethanol/week)**							
Non-drinker	8,779	71,236	4	(17.4)	1.00	Ref	
<10 g/week	14,137	115,198	11	(47.8)	2.01	0.63	6.39
10–30 g/week	10,142	83,193	7	(30.4)	1.75	0.50	6.12
>30 g/week	7,467	60,706	‡	(4.4)	0.33	0.04	3.00
P trend					0.173		
Continuous, per 1 SD					0.68	0.45	1.01

GC, gastric cancer; HR, hazard ratio; CI, confidence interval; SD, standard drink.
[†]Adjusted for education, smoking status, marital status, family history of gastric cancer, exercise, BMI, and total energy intake.
[‡]Frequencies <5 not shown.

Table S3 Hazard ratios and 95% confidence intervals of non-cardia gastric cancer (n = 126,973) according to alcohol consumption

	Men								Women						
Variables	Number of participants	Person-years	GC	(%)	HR†	95% CI		Variables	Number of participants	Person-years	GC	(%)	HR†	95% CI	
**Drink status**								**Drink status**							
Never	8,859	71,696	84	(0.9)	1.00	Ref		Never	58,119	47,248	267	(0.5)	1.00	Ref	
Ever	32,964	269,562	356	(1.1)	1.31	1.02	1.67	Ever	27,031	221,629	101	(0.4)	1.02	0.81	1.29
**Drink duration (years)**								**Drink duration (years)**							
Non-drinker	8,859	71,696	84	(0.9)	1.00	Ref		Non-drinker	58,119	47,248	267	(0.5)	1.00	Ref	
≤20 years	7,677	64,362	55	(0.7)	1.39	0.97	1.99	≤10 years	8,746	72,912	29	(0.3)	0.86	0.59	1.27
21–30 years	13,207	110,564	124	(0.9)	1.42	1.06	1.91	11–20 years	9,355	75,233	30	(0.3)	0.95	0.65	1.4
31–40 years	8,623	67,554	121	(1.4)	1.27	0.96	1.70	≥21 years	7,484	59,130	38	(0.5)	1.19	0.85	1.67
≥41 years	2,774	20,820	51	(1.8)	1.17	0.82	1.69	*P* trend					0.507		
P trend					0.089										
**Drink frequency (times/week)**								**Drink frequency (times/week)**							
Non-drinker	8,859	71,696	84	(0.9)	1.00	Ref		Non-drinker	58,119	47,248	267	(0.5)	1.00	Ref	
≤1 time/week	9,787	79,460	100	(1.0)	1.26	0.95	1.68	<1 time/week	7,411	60,379	18	(0.2)	0.92	0.57	1.49
2–3 times/week	11,074	90,918	104	(0.9)	1.20	0.90	1.60	1 time/week	11,652	95,390	48	(0.4)	1.12	0.82	1.52
4 times/week	7,011	56,822	76	(1.1)	1.41	1.03	1.93	≥2 times/week	6,765	54,501	30	(0.4)	0.84	0.57	1.22
≥5 times/week	4,167	33,486	63	(1.5)	1.7	1.23	2.36	*P* trend					0.749		
P trend					0.003										
**Ethanol intake (g ethanol/week)**								**Ethanol intake (g ethanol/week)**							
Non-drinker	8,859	71,696	84	(0.9)	1.00	Ref		Non-drinker	58,119	47,248	267	(0.5)	1.00	Ref	
<10 g/week	14,274	116,011	144	(1.0)	1.23	0.94	1.6	≤10 g/week	7,929	64,780	32	(0.4)	0.99	0.68	1.42
10–30 g/week	10,246	83,782	110	(1.1)	1.38	1.04	1.84	10–20 g/week	8,897	72,789	22	(0.2)	1.02	0.66	1.57
>30 g/week	7,556	61,239	90	(1.2)	1.57	1.16	2.12	>20 g/week	9,021	72,851	19	(0.2)	1.04	0.65	1.66
P trend					0.006			*P* trend					0.490		
Continuous, per 1 SD					1.00	0.99	1.01	Continuous, per 1 SD					0.81	0.60	1.10

GC, gastric cancer; HR, hazard ratio; CI, confidence interval; SD, standard drink.
†Adjusted for education, smoking status, marital status, family history of gastric cancer, exercise, BMI, and total energy intake.

Table S4 Hazard ratios and 95% confidence intervals of gastric cancer according to alcohol consumption (including former drinkers)

Variables	Men (n = 44,809)							Variables	Women (n = 87,440)						
	No. of participants	Person-years	GC	(%)	HR[†]	95% CI			No. of participants	Person-years	GC	(%)	HR[†]	95% CI	
**Drink status**								**Drink status**							
Non-drinker	8,968	77,924	87	(1.0)	1.00	Ref		Non-drinker	58,825	515,102	283	(0.5)	1.00	Ref	
Former drinker	3,150	27,832	31	(1.0)	0.94	0.62	1.42	Former drinker	1,768	16,411	9	(0.5)	1.17	0.60	2.28
Drinker	33,184	287,973	375	(1.1)	1.31	1.03	1.67	Drinker	27,241	235,840	102	(0.4)	1.01	0.80	1.28
**Drink duration**								**Drink duration**							
Non-drinker	8,968	77,924	87	(1.0)	1.00	Ref		Non-drinker	58,825	515,102	283	(0.5)	1.00	Ref	
≤20 years	8,950	77,723	69	(0.8)	1.30	0.94	1.81	<11 years	3,757	34,142	16	(0.4)	1.11	0.67	1.84
≥21 years	26,428	228,722	330	(1.3)	1.29	1.01	1.64	≥11 years	23,260	197,221	84	(0.4)	1.00	0.78	1.28
P trend					0.040			*P* trend					0.968		
**Drink frequency**								**Drink frequency**							
Non-drinker	8,968	77,924	87	(1.0)	1.00	Ref		Non-drinker	58,825	515,102	283	(0.5)	1.00	Ref	
≤1 time/week	9,900	85,490	113	(1.1)	1.31	0.99	1.74	<1 time/week	7,496	68,661	27	(0.4)	0.93	0.63	1.39
2–3 times/week	11,222	97,125	111	(1.0)	1.18	0.89	1.57	1 time/week	11,790	77,883	47	(0.4)	1.09	0.80	1.49
4 times/week	7,117	61,141	78	(1.1)	1.34	0.98	1.83	≥2 times/week	6,852	77,215	22	(0.3)	0.93	0.60	1.45
≥5 times/week	4,247	36,605	65	(1.5)	1.62	1.17	2.25	*P* trend					0.947		
P trend					0.017										
**Ethanol intake[‡]**								**Ethanol intake[‡]**							
Non-drinker	8,968	77,924	87	(1.0)	1.00	Ref		Non-drinker	58,825	515,102	283	(0.5)	1.00	Ref	
<10 g/day	14,373	123,873	159	(1.1)	1.25	0.96	1.63	≤10 g/week	7,990	68,661	32	(0.4)	1.00	0.69	1.44
10–30 g/day	10,315	89,243	118	(1.1)	1.37	1.03	1.83	10–30 g/week	8,972	77,883	36	(0.4)	1.09	0.77	1.56
>30 g/day	7,602	65,621	91	(1.2)	1.47	1.09	1.99	>30 g/week	9,086	77,215	28	(0.3)	0.92	0.62	1.37
P trend					0.033			*P* trend					0.742		
Continuous, per 1 SD					1.000	0.997	1.002	Continuous, per 1 SD					0.87	0.647	1.167

GC, gastric cancer; HR, hazard ratio; CI, confidence interval; SD, standard drink.
[†]Adjusted for smoking status, education, marital status, family history of gastric cancer, exercise, BMI, and total energy intake.
[‡]Ethanol intake refers to the amount of pure alcohol found in all alcohol beverages consumed by study participants, converted to grams per day or per week.

Table S5 Hazard ratios and 95% confidence intervals of gastric cancer according to alcohol consumption among total participants

Variables	Total (n = 128,218)						
	n	Person-years	GC	(%)	HR[†]	95% CI	
**Status**							
Non-drinker	67,793	593,026	370	0.6	1.00	Ref	
Drinker	60,425	523,813	477	0.8	1.15	0.98	1.35
**Duration**							
Non-drinker	67,793	593,026	370	0.6	1.00	Ref	
<21 years	25,949	224,387	118	0.5	1.08	0.87	1.34
≥21 years	32,330	277,235	349	1.1	1.24	1.03	1.49
P trend					0.04		
**Frequency**							
Non-drinker	67,793	593,026	370	0.6	1.00	Ref	
≤1 time/week	26,070	224,927	179	0.7	1.17	0.97	1.41
2–3 times/week	18,109	156,007	135	0.8	1.06	0.85	1.33
4 times/week	9,135	78,183	82	0.9	1.16	0.89	1.51
≥5 times/week	4,970	42,848	66	1.3	1.44	1.08	1.92
P trend					0.05		
**Amount of ethanol**[‡]							
Non-drinker	67,793	593,026	370	0.6	1.00	Ref	
<10 g/week	36,578	314,900	247	0.7	1.13	0.95	1.35
10–30 g/week	13,259	114,368	124	0.9	1.19	0.94	1.50
>30 g/week	8,501	73,229	93	1.1	1.32	1.01	1.71
P trend					0.07		
Continuous, per 1 SD					1.00	1.00	1.00

GC, gastric cancer; HR, hazard ratio; CI, confidence interval; SD, standard drink.
[†]Adjusted for education, smoking status, marital status, family history of gastric cancer, exercise, BMI, and total energy intake.
[‡]Amount of ethanol refers to the amount of pure alcohol found in all alcohol beverages consumed by study participants, converted to grams per week.

Table S6 Association between alcohol consumption pattern and gastric cancer in men, stratified by alcohol intake duration

Consumption pattern	Frequency	Amount of ethanol	*n*	Person-years	GC	(%)	HR†	95% CI	
**Drink duration <21 years**									
Non-drinker			8,968	77,924	87	(1.0)	0.80	0.54	1.19
Infrequent-light	<5 times/week	<40 g ethanol/day	6,299	54,615	43	(0.7)	1.00	Ref	
Infrequent-heavy	<5 times/week	≥40 g ethanol/day	196	1,655	‡	(<0.1)	2.23	0.95	5.26
Frequent-light	≥5 times/week	<40 g ethanol/day	344	2,910	6	(1.7)	1.43	0.44	4.62
Frequent-heavy	≥5 times/week	≥40 g ethanol/day	303	2,618	3	(1.0)	0.96	0.63	1.48
**Drink duration ≥21 years**									
Non-drinker			8,968	77,924	87	(1.0)	0.76	0.59	0.98
Infrequent-light	<5 times/week	<40 g ethanol/day	19,115	164,610	235	(1.2)	1.00	Ref	
Infrequent-heavy	<5 times/week	≥40 g ethanol/day	875	7,510	12	(1.4)	1.31	0.73	2.34
Frequent-light	≥5 times/week	<40 g ethanol/day	1,588	13,532	29	(1.8)	1.35	0.92	1.99
Frequent-heavy	≥5 times/week	≥40 g ethanol/day	1,840	16,052	24	(1.3)	1.01	0.66	1.53

GC, gastric cancer; HR, hazard ratio; CI, confidence interval.
†Adjusted for education, smoking status, marital status, family history of gastric cancer, exercise, BMI, and total energy intake.
‡Frequencies <5 not shown.

Table S7 Hazard ratios and 95% confidence intervals of gastric cancer according to alcohol consumption among green tea drinkers

Variables	Men: Number of participants	Person-years	GC	(%)	HR†	95% CI		Variables	Women: Number of participants	Person-years	GC	(%)	HR†	95% CI	
**Drink status**								**Drink status**							
Never	5,049	45,255	52	1.0	1.00	Ref		Never	33,441	305,765	178	(0.5)	1.00	Ref	
Ever	20,849	185,116	248	1.2	1.34	0.99	1.83	Ever	16,891	152,115	64	(0.4)	0.90	0.67	1.21
**Drink duration (years)**								**Drink duration (years)**							
Non-drinker	5,049	45,255	52	1.0	1.00	Ref		Non-drinker	33,441	305,765	178	0.5	1.00	Ref	
<21 years	5,038	44,726	36	0.7	1.26	0.81	1.96	<11 years	2,227	21,067	12	0.5	1.22	0.68	2.20
≥21 years	15,378	136,115	209	1.4	1.38	1.01	1.89	≥11 years	13,626	119,751	50	0.4	0.90	0.65	1.24
P trend					0.04			*P* trend					0.52		
**Drink frequency (times/week)**								**Drink frequency (times/week)**							
Non-drinker	5,049	45,255	52	1.0	1.00	Ref		Non-drinker	33,441	305,765	178	0.5	1.00	Ref	
≤1 time/week	5,989	52,816	75	1.3	1.39	0.97	2.00	<1 time/week	4,572	40,737	17	0.4	0.86	0.52	1.42
2–3 times/week	7,107	63,089	77	1.1	1.25	0.87	1.79	1 time/week	7,338	66,172	29	0.4	0.96	0.64	1.43
4 times/week	4,687	41,262	52	1.1	1.32	0.89	1.95	≥2 times/week	4,141	36,523	14	0.3	0.86	0.49	1.49
≥5 times/week	2,457	21,647	38	1.6	1.62	1.06	2.49	*P* trend					0.584		
P trend					0.123										
**Ethanol intake (g ethanol/week)**								**Ethanol intake (g ethanol/week)**							
Non-drinker	5,049	45,255	52	1.0	1.00	Ref		Non-drinker	33,441	305,765	178	0.5	1.00	Ref	
<10 g/week	8,973	79,039	115	1.3	1.39	0.99	1.93	≤10 g/week	4,916	43,927	20	0.4	0.91	0.57	1.45
10–30 g/week	6,571	58,302	67	1.0	1.19	0.82	1.72	10–20 g/week	5,640	50,859	21	0.4	0.91	0.57	1.43
>30 g/week	4,722	41,751	60	1.3	1.53	1.04	2.25	>20 g/week	5,505	48,735	19	0.4	0.90	0.55	1.46
P trend					0.150			*P* trend					0.634		
Continuous, per 1 SD					1.00	0.96	1.03	Continuous, per 1 SD					0.95	0.71	1.28

GC, gastric cancer; HR, hazard ratio; CI, confidence interval; SD, standard drink.
†Adjusted for education, smoking status, marital status, family history of gastric cancer, exercise, BMI, and total energy intake.
